# Hospital Use of a Web-Based Clinical Knowledge Support System and In-Training Examination Performance Among Postgraduate Resident Physicians in Japan: Nationwide Observational Study

**DOI:** 10.2196/52207

**Published:** 2024-05-30

**Authors:** Koshi Kataoka, Yuji Nishizaki, Taro Shimizu, Yu Yamamoto, Kiyoshi Shikino, Masanori Nojima, Kazuya Nagasaki, Sho Fukui, Sho Nishiguchi, Kohta Katayama, Masaru Kurihara, Rieko Ueda, Hiroyuki Kobayashi, Yasuharu Tokuda

**Affiliations:** 1 Division of Medical Education, Juntendo University School of Medicine, Tokyo, Japan; 2 Department of Diagnostic and Generalist Medicine, Dokkyo Medical University Hospital, Tochigi, Japan; 3 Division of General Medicine, Center for Community Medicine, Jichi Medical University, Tochigi, Japan; 4 Department of Community-Oriented Medical Education, Chiba University Graduate School of Medicine, Chiba, Japan; 5 Center for Translational Research, The Institute of Medical Science Hospital, The University of Tokyo, Tokyo, Japan; 6 Department of Internal Medicine, Mito Kyodo General Hospital, University of Tsukuba, Ibaraki, Japan; 7 Department of Emergency and General Medicine, Kyorin University, Tokyo, Japan; 8 Department of General Internal Medicine, Shonan Kamakura General Hospital, Kanagawa, Japan; 9 Division of General Internal Medicine, St. Marianna University School of Medicine, Kanagawa, Japan; 10 Department of Clinical Epidemiology, Graduate School of Medicine, Fukushima Medical University, Fukushima, Japan; 11 Department of Patient Safety, Nagoya University Hospital, Nagoya, Japan; 12 Medical Technology Innovation Center, Juntendo University, Tokyo, Japan; 13 Muribushi Okinawa Center for Teaching Hospitals, Okinawa, Japan; 14 Tokyo Foundation for Policy Research, Tokyo, Japan

**Keywords:** clinical knowledge support system, GM-ITE, postgraduate clinical resident, in-training examination performance, exam, exams, examination, examinations, resident, residents, cross-sectional, national, nationwide, postgraduate, decision support, point-of-care, UpToDate, DynaMed, knowledge support, medical education, performance, information behavior, information behaviour, information seeking, teaching, pedagogy, pedagogical, log, logs, usage, evidence-based medicine, EBM, educational, decision support system, clinical decision support, Japan, General Medicine In-Training Examination

## Abstract

**Background:**

The relationship between educational outcomes and the use of web-based clinical knowledge support systems in teaching hospitals remains unknown in Japan. A previous study on this topic could have been affected by recall bias because of the use of a self-reported questionnaire.

**Objective:**

We aimed to explore the relationship between the use of the Wolters Kluwer UpToDate clinical knowledge support system in teaching hospitals and residents’ General Medicine In-Training Examination (GM-ITE) scores. In this study, we objectively evaluated the relationship between the total number of UpToDate hospital use logs and the GM-ITE scores.

**Methods:**

This nationwide cross-sectional study included postgraduate year–1 and –2 residents who had taken the examination in the 2020 academic year. Hospital-level information was obtained from published web pages, and UpToDate hospital use logs were provided by Wolters Kluwer. We evaluated the relationship between the total number of UpToDate hospital use logs and residents’ GM-ITE scores. We analyzed 215 teaching hospitals with at least 5 GM-ITE examinees and hospital use logs from 2017 to 2019.

**Results:**

The study population consisted of 3013 residents from 215 teaching hospitals with at least 5 GM-ITE examinees and web-based resource use log data from 2017 to 2019. High-use hospital residents had significantly higher GM-ITE scores than low-use hospital residents (mean 26.9, SD 2.0 vs mean 26.2, SD 2.3; *P*=.009; Cohen *d*=0.35, 95% CI 0.08-0.62). The GM-ITE scores were significantly correlated with the total number of hospital use logs (Pearson *r*=0.28; *P*<.001). The multilevel analysis revealed a positive association between the total number of logs divided by the number of hospital physicians and the GM-ITE scores (estimated coefficient=0.36, 95% CI 0.14-0.59; *P*=.001).

**Conclusions:**

The findings suggest that the development of residents’ clinical reasoning abilities through UpToDate is associated with high GM-ITE scores. Thus, higher use of UpToDate may lead physicians and residents in high-use hospitals to increase the implementation of evidence-based medicine, leading to high educational outcomes.

## Introduction

Sir William Osler [1] stated that “to study the phenomena of disease without books is to sail in an uncharted sea, while to study books without patients is not to go to sea at all.” Self-learning is known to develop basic clinical skills [2-4], and several studies have demonstrated the effectiveness of web-based clinical knowledge support systems. For example, a study examining the US Residency Internal Medicine In-Training Examination (IM-ITE) score reports a 3.7% increase in the IM-ITE score per 100 hours of UpToDate use [5]. In addition, UpToDate users are more satisfied with their answer accuracy, interaction, and overall satisfaction than PubMed Clinical Queries users [6]. Thus, UpToDate may be effective at the hospital level because hospitals using UpToDate have been reported to show a significantly shorter length of stay for patients [7-9]. UpToDate is already the most widely used web-based clinical knowledge support system among residents (65.5%) and the third most used system among physicians (40.4%) [9].

The General Medicine In-Training Examination (GM-ITE) is an in-training examination developed to provide residents and training program directors with an objective, reliable, and valid assessment of clinical knowledge during training. It uses the same methodology as the IM-ITE [10-12] and comprises the following 4 domains: medical interview/professionalism, symptomatology/clinical reasoning, clinical procedures, and disease knowledge. The examinations consist of 60 questions (6 on medical interview/professionalism, 15 on symptomatology/clinical reasoning, 15 on clinical procedure, and 24 on disease knowledge) and include video- and audio-format questions. The GM-ITE was first introduced in 2011 by the Japan Institute for Advancement of the Medical Education Program (JAMEP), a nonprofit organization, and is administered annually. The questions are prepared annually by a committee of experienced physicians, and peers are reviewed by an independent committee. The examinations are open to the residents of teaching hospitals that have applied to offer the examinations [13,14].

We previously reported that self-study time and use of UpToDate had positive relationships with GM-ITE scores [4]. However, those findings could have been affected by recall bias because of the use of a self-reported questionnaire, which meant that objectivity could not be guaranteed. In this study, therefore, we objectively evaluated the relationship between the total number of hospital use logs in UpToDate and the GM-ITE scores of hospital residents.

Hospital use logs were used because residents have several opportunities to acquire second-hand knowledge from their supervisors, reflecting the evidence-based medicine (EBM) culture of teaching hospitals. The introduction of clinical knowledge support systems has recently increased among resident and senior doctors, although the frequency of use is low because of language barriers and is far from the global standard [9]. The postgraduate 2-year residency system was established in 2004 in Japan. The use of the *Yanegawara* (“tiled roof” in Japanese) style of education, in which senior doctors teach resident physicians and postgraduate year (PGY)–2 residents teach PGY-1 residents based on EBM using web-based medical resources, such as UpToDate, has also become widespread [15]. The merit of the *Yanegawara*-style education is the aspect of teaching among residents with close grade levels. Internationally, peer teaching or peer tutor systems have been shown to be effective in medical education [16,17].

The aim of this study was to evaluate the correlation between the total number of UpToDate hospital use logs and the GM-ITE scores of resident physicians objectively.

## Methods

### Study Design and Population

We conducted a nationwide observational study of postgraduate residents in Japan using both mean GM-ITE scores and the total number of UpToDate hospital use logs to examine their relationship. The 2020 GM-ITE and self-reported questionnaire were conducted between January 13 and 31, 2021, and the data were collected during the same period. We accessed the data set for research purposes on June 16, 2021.

In Japan, postgraduate resident physicians are required to undergo at minimum a 2-year postgraduate residency program after 6 years of undergraduate medical school. In the program, the resident physicians rotate around 7 clinical departments: internal medicine, surgery, emergency medicine, pediatrics, obstetrics and gynecology, psychiatry, and community-based medicine. The Ministry of Health, Labour and Welfare has established guidelines for postgraduate clinical training programs to teach communication skills, professionalism, and ethics, in addition to basic clinical knowledge and skills, to resident physicians. Medical students in their final year of an undergraduate medical program can apply for the postgraduate residency program at more than approximately 1000 clinical teaching hospitals in Japan using a web-based matching system [18].

### Measurements

We collected hospital-level information (number of physicians, monthly salary, number of ambulances, number of permitted beds, type of tertiary emergency care, location, and type of hospital) from published web pages. The hospital use logs of the web-based clinical knowledge support system (UpToDate) in the 3 years from 2017 to 2019 were provided by Wolters Kluwer. UpToDate log data were defined as the number of topic review page views. We also collected GM-ITE scores. We hypothesized that supervisors’ use of UpToDate reflects the culture of EBM resident education at each teaching hospital. Furthermore, we decided to use UpToDate hospital use logs from 2017 to 2019 to examine their association with the 2020 GM-ITE scores because educational effects are not immediately reflected after an intervention. Resident-level information (sex, grade, number of monthly emergency department duties, average number of patients in charge, general medicine department rotation, self-study time, and weekly duty hours) were obtained using a self-reported questionnaire administered immediately after the GM-ITE. These variables were selected based on previous studies [19-21].

### Statistical Analyses

Hospitals were classified as low or high use according to their UpToDate hospital use logs. The total number of use logs was divided by the number of physicians and was log-transformed into base 2. The monthly salary, number of ambulances, and number of permitted beds were also log-transformed into base 2. Low-use hospitals were defined as those with fewer than the median log-transformed number of hospital use logs, whereas high-use hospitals were defined as those with greater than or equal to the median log-transformed number of hospital use logs. Differences between low-use and high-use hospitals were examined for statistical significance using the Student 2-tailed *t* test. Categorical variables were compared using the *χ*^2^ test and presented as frequencies with percentages. The effect size (Cohen *d*) was estimated using the median difference between low- and high-use hospitals divided by the pooled SD—a value of 0.2 was considered a small effect, 0.5 was considered a medium effect, and 0.8 was considered a large effect [22]. Hospital-level analysis was performed using scatter plots to examine the association between the mean GM-ITE score and the number of UpToDate hospital use logs aggregated at the hospital level. We analyzed the association between the GM-ITE scores and the total number of UpToDate hospital use logs in each hospital over 3 years, using a linear multilevel regression model. The multilevel analysis was adjusted for sex, location, and type of hospital in addition to statistically significant factors in the univariate analysis. In those analyses, the domain of medical interview/professionalism in the GM-ITE was excluded from the analysis because we believed that it was not a clinical skill that could be improved using UpToDate. All analyses were performed using SAS software (version 9.4; SAS Institute Inc).

### Ethical Considerations

This study was performed in accordance with the principles of the Declaration of Helsinki and STROBE (Strengthening the Reporting of Observational Studies in Epidemiology) guidelines. All the methods followed the *Ethical Guidelines for Medical and Health Research Involving Human Subjects*. Informed consent was obtained from each participant after clarifying the explanatory research document, including data anonymization and voluntary participation. Only participants who provided consent were included in this study, and they were also provided an opportunity to opt out. The study was approved by the Ethics Review Board of JAMEP (approval 21-1).

## Results

The 2020 GM-ITE was offered at 593 teaching hospitals nationwide, and 7669 residents took the exams. A total of 6816 residents from 588 teaching hospitals participated in the survey on the training environment. The study population consisted of 3013 residents from 215 teaching hospitals with at least 5 GM-ITE examinees and web-based resource use log data from 2017 to 2019. Hospitals in all regions of Japan, namely Hokkaido, Tohoku, Kanto, Chubu, Kinki, Chugoku, Shikoku, Kyushu, and Okinawa, were included. The mean number of GM-ITE examinees per hospital was 14.1 (SD 8.6).

The hospital-level information is presented in Table 1. The mean GM-ITE score of all the hospitals was 26.5 (SD 2.2); of the 215 hospitals, 115 (53.5%) were secondary care hospitals, 159 (74%) were located in rural areas, and 204 (94.9%) were community-based hospitals. Residents of high-use hospitals achieved significantly higher GM-ITE scores than those of low-use hospitals (mean 26.9, SD 2.0 vs mean 26.2, SD 2.3; *P*=.009; Cohen *d*=0.35, 95% CI 0.08-0.62). Monthly salary (in JPY ¥100,000; JPY ¥100=US $0.64) was significantly higher in low-use hospitals than high-use hospitals (mean 3.7, SD 0.8 vs mean 3.3, SD 0.7; *P*<.001). The resident-level information is presented in Multimedia Appendix 1; 68.5% (2076/3031) were male and 50.5% (1531/3031) were PGY-2 residents.

**Figure 1.**
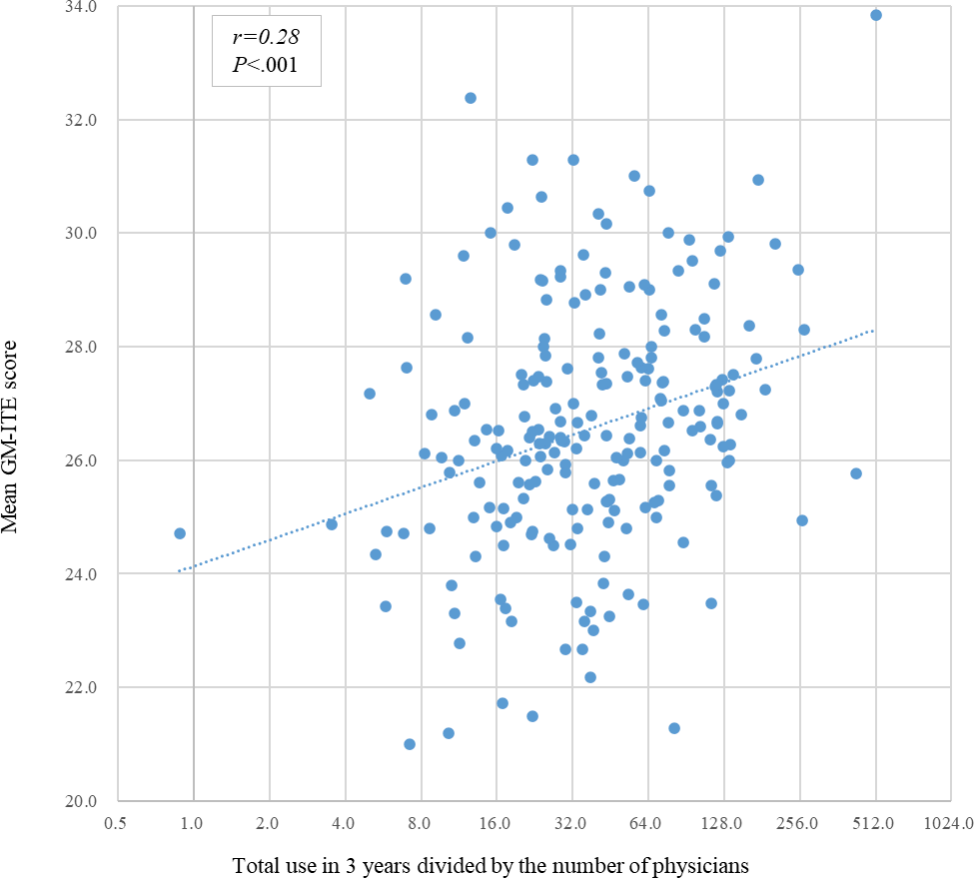
Correlation between total use of UpToDate and mean General Medicine In-Training Examination (GM-ITE) scores.

Correlations between total use in 3 years divided by the number of physicians and GM-ITE scores were analyzed (Figure 1). The mean GM-ITE hospital score was significantly correlated with the total number of UpToDate hospital use logs (Pearson *r*=0.28, *P*<.001; Spearman *r*=0.27, *P*<.001). The linear regression function was *y = 24.13 + 0.66* × *log*_*2*_*(total use/number of physicians)*; therefore, the difference in the mean GM-ITE score between the total use divided by the number of physicians at values of 8 and 128 was 2.64 (Figure 1). Multimedia Appendix 2 shows the relationship between GM-ITE scores and hospital- and resident-level information using an univariate analysis. The statistically significant factors were log-transformed total number of hospital use logs in 3 years divided by the number of physicians (*P*<.001), log-transformed number of ambulances (*P*<.001), log-transformed number of permitted beds (*P=*.005), type of tertiary emergency care (*P*=.01), grade (*P*<.001), number of monthly emergency department duty (*P*=.004-.046), average number of patients in charge (from *P*<.001 to *P*=.01), general medicine department rotation (*P*=.004), self-study time (*P*=.02-.04), and weekly duty hours (*P*<.001). The multilevel analysis was adjusted for all these factors in addition to sex, location, and type of hospital. Table 2 shows the relationship between GM-ITE scores and hospital- and resident-level information using a multilevel analysis. The multilevel analysis revealed a positive association between 3-year total hospital use logs and GM-ITE scores (estimated coefficient=0.36, 95% CI 0.14-0.59; *P*=.001). Multimedia Appendix 3 shows the results of the analysis of all 4 domains (medical interview/professionalism, symptomatology/clinical reasoning, clinical procedures, and disease knowledge). The result also revealed a positive association between the use of UpToDate and GM-ITE scores (estimated coefficient=0.41, 95% CI 0.18-0.65; *P*<.001).

**Table 1.** Background characteristics of the teaching hospitals.

Hospital-level information		Total (N=215)	Low-use hospitals (n=107)	High-use hospitals (n=108)	*P* value
Total number of use logs of UpToDate, mean (SD)		10,485.1 (20,231.4)	2578.7 (2,278.2)	18,318.2 (26,249.5)	<.001
Number of physicians, mean (SD)		144.2 (91.4)	116.3 (61.9)	171.8 (106.6)	<.001
Total use in 3 years/number of physicians, mean (SD)		56.5 (62.2)	20.2 (8.7)	92.4 (71.1)	<.001
Log-transformed total use in 3 years/number of physicians, mean (SD)		5.2 (1.3)	4.1 (0.9)	6.3 (0.8)	<.001
Monthly salary (in JPY ¥100,000^a^), mean (SD)		3.5 (0.8)	3.7 (0.8)	3.3 (0.7)	<.001
Log-transformed monthly salary (in JPY ¥100,000), mean (SD)		1.8 (0.3)	1.9 (0.3)	1.7 (0.3)	<.001
Number of ambulances, mean (SD)		4882.6 (2399.2)	4462.0 (2183.9)	5299.4 (2536.8)	.01
Log-transformed number of ambulances, mean (SD)		12.1 (0.8)	11.9 (0.7)	12.2 (0.8)	.02
Number of permitted beds, mean (SD)		497.8 (166.8)	461.2 (158.9)	534.0 (167.3)	.001
Log-transformed number of permitted beds, mean (SD)		8.9 (0.5)	8.8 (0.5)	9.0 (0.4)	<.001
**Type of tertiary emergency care, n (%)**					.02
	Tertiary medical care	100 (46.5)	41 (38.3)	59 (54.6)	
	Secondary care	115 (53.5)	66 (61.7)	49 (45.4)	
**Location, n (%)**					.13
	Urban area	56 (26)	23 (21.5)	33 (30.6)	
	Rural area	159 (74)	84 (78.5)	75 (69.4)	
**Type of hospital, n (%)**					.03
	University hospital	11 (5.1)	2 (1.9)	9 (8.3)	
	Community-based hospital	204 (94.9)	105 (98.1)	99 (91.7)	
GM-ITE^b^ score, mean (SD)		26.5 (2.2)	26.2 (2.3)	26.9 (2.0)	.009

a JPY ¥100=US $0.64.

b GM-ITE: General Medicine In-Training Examination.

**Table 2.** Factors related to General Medicine In-Training Examination (GM-ITE) scores (multilevel analysis).

Factors			Estimated coefficient (95% CI)			*P* value
**Hospital-level information**						
	Log-transformed total use of UpToDate in 3 years/number of physicians		0.36 (0.14 to 0.29)			.001
	Log-transformed number of ambulances		0.34 (−0.08 to 0.77)			.11
	Log-transformed number of permitted beds		0.36 (−0.39 to 1.12)			.35
	**Type of tertiary emergency care**					
		Tertiary medical care	Reference			Reference
		Secondary care	−0.20 (−0.80 to 0.40)			.51
	**Location**					
		Urban area	Reference			Reference
		Rural area	0.72 (0.09 to 1.35)			.02
	**Type of hospital**					
		University hospital	Reference			Reference
		Community-based hospital	0.52 (−0.82 to 1.88)			.44
**Resident-level information**						
	**Sex**					
		Male	Reference			Reference
		Female	0.08 (−0.28 to 0.45)			.66
	**Grade**					
		PGY^a^-1	Reference			Reference
		PGY-2	0.81 (0.45 to 1.17)			<.001
	**Number of monthly emergency department duties**					
		0	Reference			Reference
		1-2	0.46 (−0.64 to 1.58)			.41
		3-5	0.81 (−0.28 to 1.92)			.15
		>6	0.48 (−0.75 to 1.72)			.45
		Unknown	−0.42 (−3.38 to 2.54)			.78
	**Average number of patients in charge**					
		0-4	Reference			Reference
		5-9	0.76 (0.30 to 1.21)			.001
		10-14	0.62 (−0.10 to 1.35)			.09
		>15	1.20 (−0.07 to 2.47)			.06
		Unknown	−1.03 (−2.27 to 0.20)			.10
	**General medicine department rotation**					
		Yes	Reference			Reference
		No	−0.12 (−0.54 to 0.29)			.56
	**Self-study time (min/d)**					
		None	Reference			Reference
		0-30	−0.10 (−1.13 to 0.92)			.84
		31-60	0.31 (−0.70 to 1.33)			.54
		61-90	0.94 (−0.12 to 2.01)			.08
		>91	1.03 (−0.25 to 2.32)			.12
	**Weekly duty hours (h/wk)**					
		0-59	Reference			Reference
		60-79	0.67 (0.27 to 1.07)			<.001
		>80	−0.10 (−0.60 to 0.38)			.67

a PGY: postgraduate year.

## Discussion

We objectively evaluated the relationship between hospital use logs of the web-based clinical knowledge support system, UpToDate, at teaching hospitals and residents’ GM-ITE scores. Residents of high-use hospitals achieved significantly higher GM-ITE scores, an objective index of the basic clinical ability of residents, than those of low-use hospitals. There are 2 main situations in which residents use web-based clinical knowledge support systems such as UpToDate: “actual clinical sittings such as bedside and outpatient care” and “self-improvement.” UpToDate is useful in situations where immediate evidence-based care must be provided to the patient in front of a resident [23]. In terms of self-improvement, among both residents and senior doctors, web-based clinical knowledge support systems could lead to the development of basic clinical abilities because they can collect the latest information more efficiently than from textbooks [9].

The use of a web-based clinical knowledge support system is associated with high GM-ITE scores owing to the residents’ knowledge of clinical reasoning. The clinical training guidelines of the Ministry of Health, Labour and Welfare highlight the importance of studying clinical reasoning and problem-solving abilities, and residents are required to have the ability to (1) make a differential diagnosis and initial response to high-frequency symptoms through an appropriate clinical reasoning process and (2) collect patient information and make clinical decisions in consideration of the patient’s intentions and quality of life based on the latest medical knowledge. Residents constantly acquire the latest medical knowledge and use evidence-based and their own experiences to solve clinical problems. The questions in the GM-ITE include clinical reasoning questions in accordance with the guidelines of the Ministry of Health, Labour and Welfare. As UpToDate contains a series of flows that “list differential diagnoses from symptoms and link them to accurate diagnoses,” we believe the use of UpToDate would help residents develop their clinical reasoning abilities. Therefore, we speculate that the development of residents’ clinical reasoning abilities through UpToDate is associated with high GM-ITE scores.

Japanese residents are required to gain greater outpatient clinical experience to acquire basic clinical skills, including communication and clinical reasoning, during this 2-year training period. The postgraduate clinical residency system has been revised regularly, and the latest revision in 2020 requires a 1-month general outpatient training rotation for residents. Therefore, outpatient training is becoming increasingly important in Japanese clinical resident education programs.

Previous studies have demonstrated the usefulness of web-based clinical knowledge support systems in outpatient care. A comparison of outpatient diagnostic errors made by physicians with and without the use of UpToDate shows that diagnostic errors were significantly reduced in the case of physicians who used UpToDate [24]. Internal medicine residents’ responses to patient-specific questions encountered in outpatient settings have been known to improve their clinical skills and patient care decisions. UpToDate has been reported to be the second most commonly used tool for gathering information after MEDLINE and is an extremely helpful information source [25]. We speculate that the GM-ITE includes questions regarding outpatient care that are associated with the development of clinical residents’ outpatient care abilities and high GM-ITE scores.

Factors significantly and positively associated with GM-ITE scores in the multilevel analysis results, besides the use of UpToDate, were location, PGY-2 grade, average number of patients in charge, and weekly duty hours. Residents of rural teaching hospitals may have the opportunity to examine more patients, because the number of physicians affiliated with rural teaching hospitals is lower than that with urban teaching hospitals. Consequently, they may acquire greater clinical experience and knowledge [26]. Regarding the difference in grades, we believe that the difference in clinical experience is directly reflected in GM-ITE scores. This finding is consistent with the results of our previous study [4]. We recommend that residents develop a proactive attitude toward patient care because basic clinical skills tend to develop with daily patient management. The results of the multilevel analysis showed that 60-79 weekly duty hours were significantly and positively associated with GM-ITE scores. This finding supports our previous results [20]; we believe there are optimal working hours for improving clinical competency.

The development of residents’ basic clinical skills does not require many supervisors; however, high-quality and highly productive education is necessary. A previous Japanese study showed that education delivered by a limited number of supervisors was more likely to develop residents’ basic clinical skills [27]. Furthermore, residents who rotated in general medicine achieved higher GM-ITE scores [4]. We believe the following factors are required for future residency education: generalist residency education by general medicine specialists; use of productive web-based clinical knowledge support systems, such as UpToDate; EBM culture; and the *Yanegawara*-style educational system.

This study has a few limitations. First, the scores were examined among a limited number of GM-ITE examinees. Although there are approximately 18,000 PGY-1 and -2 residents in Japan, only 3013 residents were analyzed in this study, accounting for approximately one-sixth (16.7%) of the total population. In addition, as the GM-ITE is a voluntary examination, a bias toward highly motivated residents taking the exam may exist. Therefore, the generalizability of this study is not ensured. Second, causal relationships could not be guaranteed because the study design was cross-sectional. To control for selection bias and to assess causality, we believe that planning a randomized controlled trial targeting nationwide resident physicians is necessary. In this randomized controlled trial, the GM-ITE scores would be the primary end point, and the intervention would control for the presence or absence of web-based clinical knowledge support systems. Third, we did not assess the baseline clinical skills of the GM-ITE examinees in this study, and differences in undergraduate medical school education could have impacted the study results. Fourth, the hospital use logs did not include detailed information, such as user information and access time. It was not possible to identify user-specific information, such as residents, physicians, and co-medical professionals (eg, nurses), from the log data. Fifth, the results came from a single web-based clinical knowledge support system. Although there are other web-based clinical knowledge support systems that aid residents, physicians, and paramedical workers, we did not compare UpToDate with them. Some resident physicians in Japan may use web-based clinical knowledge support systems other than UpToDate. Although we could not obtain data on systems other than UpToDate for this study, we aim to include them in our next research project to validate the current results.

In conclusion, residents in high-use hospitals had significantly higher GM-ITE scores than those in low-use hospitals, indicating that GM-ITE scores are associated with web-based resource use logs. A previous study showed an association between web-based resource use and resident GM-ITE scores using data from a self-reported survey of clinical residents [4]. Our findings are consistent with those of previous studies and include data that ensure objectivity. Frequent use of web-based clinical knowledge support systems will increase the likelihood of physicians, including faculty, senior, and junior residents, implementing EBM and senior physicians teaching juniors using the *Yanegawara*-style education, which may lead to higher educational outcomes.

## Supplementary material

10.2196/52207 Multimedia Appendix 1 Background characteristics of the residents.

10.2196/52207 Multimedia Appendix 2 Factors related to General Medicine In-Training Examination scores (univariate analysis).

10.2196/52207 Multimedia Appendix 3 Factors related to General Medicine In-Training Examination scores, including the 4 domains (multilevel analysis).

## Abbreviations

EBM: evidence-based medicine
GM-ITE: General Medicine In-Training Examination
IM-ITE: Internal Medicine In-Training Examination
JAMEP: Japan Institute for Advancement of the Medical Education Program
PGY: postgraduate year
STROBE: Strengthening the Reporting of Observational Studies in Epidemiology
