# Supplementary material for: Hospital Use of a Web-Based Clinical Knowledge Support System and In-Training Examination Performance Among Postgraduate Resident Physicians in Japan: Nationwide Observational Study
Source: JMIR Med Educ. 2024 May 30;10:e52207. doi: 10.2196/52207 (PMC11154652; doi:10.2196/52207)
Supplement: Multimedia Appendix 1 [file mededu-v10-e52207-s001.docx]

**Multimedia Appendix 1.** Background characteristics of the residents.

|  | Total (n = 3,031) | Low-use hospitals (n = 1,504) | High-use hospitals (n = 1,527) | *P*-value |
| --- | --- | --- | --- | --- |
| Residents’ information |  |  |  |  |
| Sex, n (%) |  |  |  | 0.139 |
| Male | 2,076 (68.5) | 1,049 (69.8) | 1,027 (67.3) |  |
| Female | 955 (31.5) | 455 (30.2) | 500 (32.7) |  |
| Grade, n (%) |  |  |  | 0.595 |
| PGY 1 | 1,500 (49.5) | 737 (49) | 763 (50) |  |
| PGY 2 | 1,531 (50.5) | 767 (51) | 764 (50) |  |
| Number of monthly emergency department duties, n (%) | | | | <0.001 |
| 0 per month | 114 (3.8) | 29 (1.9) | 85 (5.6) |  |
| 1–2 per month | 346 (11.4) | 129 (8.6) | 217 (14.2) |  |
| 3–5 per month | 2,218 (73.2) | 1,175 (78.1) | 1,043 (68.3) |  |
| >6 | 342 (11.3) | 169 (11.2) | 173 (11.3) |  |
| Unknown | 11 (0.3) | 2 (0.1) | 9 (0.6) |  |
| Average number of patients in their charge, n (%) | | | | <0.001 |
| 0–4 | 711 (23.5) | 331 (22.0) | 380 (24.9) |  |
| 5–9 | 1,869 (61.7) | 927 (61.6) | 942 (61.7) |  |
| 10–14 | 295 (9.7) | 163 (10.8) | 132 (8.6) |  |
| >15 | 90 (3.0) | 33 (2.2) | 57 (3.7) |  |
| Unknown | 66 (2.1) | 50 (3.3) | 16 (1.1) |  |
| General medicine department rotation, n (%) | | | | <0.001 |
| Yes | 1,403 (46.3) | 570 (37.9) | 833 (54.6) |  |
| No | 1,628 (53.7) | 934 (62.1) | 694 (45.4) |  |
| Self-study time, n (%) |  |  |  | 0.055 |
| None | 92 (3.0) | 51 (3.4) | 41 (2.7) |  |
| 0–30 min per day | 978 (32.3) | 518 (34.4) | 460 (30.1) |  |
| 31–60 min per day | 1287 (42.5) | 617 (41.0) | 670 (43.9) |  |
| 61 to 90 min per day | 543 (17.9) | 260 (17.3) | 283 (18.5) |  |
| >91 min per day | 131 (4.3) | 58 (3.9) | 73 (4.8) |  |
| Weekly duty hour, n (%) |  |  |  | 0.020 |
| 0 to 59 hours per week | 1234 (40.7) | 625 (41.6) | 609 (39.9) |  |
| 60 to 79 hours per week | 1162 (38.3) | 595 (39.6) | 567 (37.1) |  |
| More than 80 hours per week | 635 (21.0) | 284 (18.8) | 351 (23.0) |  |
| Note: PGY: postgraduate year. | | | | |
