# Supplementary material for: Hospital Use of a Web-Based Clinical Knowledge Support System and In-Training Examination Performance Among Postgraduate Resident Physicians in Japan: Nationwide Observational Study
Source: JMIR Med Educ. 2024 May 30;10:e52207. doi: 10.2196/52207 (PMC11154652; doi:10.2196/52207)
Supplement: Multimedia Appendix 2 [file mededu-v10-e52207-s002.docx]

**Multimedia Appendix 2.** Factors related to GM-ITE score (univariate analysis).

|  | Estimated coefficient | 95% confidence interval | | *P*-value |
| --- | --- | --- | --- | --- |
| Hospital information (n = 215) |  | Lower limit | Upper limit |  |
| Log-transformed total use of UpToDate in 3 years / number of physicians | 0.46 | 0.25 | 0.67 | <0.001 |
| Log-transformed monthly salary (100,000 units) | −0.50 | −1.49 | 0.48 | 0.319 |
| Log-transformed number ambulances | 0.64 | 0.27 | 1.02 | <0.001 |
| Log-transformed number of permitted beds | 0.90 | 0.27 | 1.54 | 0.005 |
| Type of tertiary emergency care |  |  |  |  |
| Tertiary medical care | 0 |  |  |  |
| Secondary care | −0.73 | −1.32 | −0.14 | 0.014 |
| Location |  |  |  |  |
| Urban area | 0 |  |  |  |
| Rural area | 0.31 | −0.36 | 0.98 | 0.365 |
| Type of hospital |  |  |  |  |
| University hospital | 0 |  |  |  |
| Community-based hospital | 0.53 | −0.80 | 1.88 | 0.433 |
| Residents’ information (n = 3013) |  |  |  |  |
| Sex |  |  |  |  |
| Male | 0 |  |  |  |
| Female | −0.11 | −0.49 | 0.27 | 0.572 |
| Grade |  |  |  |  |
| PGY 1 | 0 |  |  |  |
| PGY 2 | 0.90 | 0.54 | 1.26 | <0.001 |
| Number of monthly emergency department duties | | | |  |
| 0 per month | 0 |  |  |  |
| 1–2 per month | 1.08 | 0.01 | 2.14 | 0.046 |
| 3–5 per month | 1.33 | 0.38 | 2.27 | 0.005 |
| >6 | 1.55 | 0.49 | 2.62 | 0.004 |
| Unknown | 0.92 | −2.18 | 4.03 | 0.560 |
| Average number of patients in their charge | | | |  |
| 0–4 | 0 |  |  |  |
| 5–9 | 1.11 | 0.68 | 1.54 | <0.001 |
| 10–14 | 0.86 | 0.18 | 1.54 | 0.012 |
| >15 | 1.78 | 0.68 | 2.88 | 0.001 |
| Unknown | −1.00 | −2.26 | 0.25 | 0.119 |
| General medicine department rotation |  |  |  |  |
| Yes | 0 |  |  |  |
| No | −0.51 | −0.87 | −0.15 | 0.004 |
| Self-study time |  |  |  |  |
| None | 0 |  |  |  |
| 0–30 min per day | −0.20 | −1.27 | 0.86 | 0.713 |
| 31–60 min per day | 0.39 | −0.66 | 1.45 | 0.460 |
| 61 to 90 min per day | 1.30 | 0.20 | 2.41 | 0.020 |
| >91 min per day | 1.43 | 0.09 | 2.76 | 0.035 |
| Weekly duty hours |  |  |  |  |
| 0–59 h per week | 0 |  |  |  |
| 60–79 h per week | 0.93 | 0.53 | 1.33 | <0.001 |
| >80 h per week | 0.45 | −0.02 | 0.93 | 0.060 |
| Note: PGY: postgraduate year. | | | | |
