# Supplementary material for: Hospital Use of a Web-Based Clinical Knowledge Support System and In-Training Examination Performance Among Postgraduate Resident Physicians in Japan: Nationwide Observational Study
Source: JMIR Med Educ. 2024 May 30;10:e52207. doi: 10.2196/52207 (PMC11154652; doi:10.2196/52207)
Supplement: Multimedia Appendix 3 [file mededu-v10-e52207-s003.docx]

**Multimedia Appendix 3.** Factors related to the GM-ITE score including the four domains (multi-level analysis).

|  | Estimated Coefficient | 95% confidence interval | | *P*-value |
| --- | --- | --- | --- | --- |
| Hospital information |  | Lower limit | Upper limit |  |
| Log-transformed total use of UpToDate in 3 years / number of physicians | 0.41 | 0.18 | 0.65 | <0.001 |
| Log-transformed number of ambulances | 0.33 | −0.12 | 0.79 | 0.153 |
| Log-transformed number of permitted beds | 0.34 | −0.47 | 1.15 | 0.410 |
| Type of tertiary emergency care |  |  |  |  |
| Tertiary medical care | 0 |  |  |  |
| Secondary care | −0.12 | −0.77 | 0.51 | 0.691 |
| Location |  |  |  |  |
| Urban area | 0 |  |  |  |
| Rural area | 0.63 | −0.03 | 1.30 | 0.064 |
| Type of hospital |  |  |  |  |
| University hospital | 0 |  |  |  |
| Community-based hospital | 0.55 | −0.89 | 2.00 | 0.451 |
| Residents’ information |  |  |  |  |
| Sex |  |  |  |  |
| Male | 0 |  |  |  |
| Female | 0.21 | −0.18 | 0.61 | 0.283 |
| Grade |  |  |  |  |
| PGY 1 | 0 |  |  |  |
| PGY 2 | 0.71 | 0.32 | 1.10 | <0.001 |
| Number of monthly emergency department duties |  |  |  |  |
| 0 per month | 0 |  |  |  |
| 1–2 per month | 0.37 | −0.81 | 1.56 | 0.535 |
| 3–5 per month | 0.77 | −0.40 | 1.95 | 0.196 |
| >6 | 0.40 | −0.92 | 1.73 | 0.549 |
| Unknown | −0.89 | −4.06 | 2.27 | 0.578 |
| Average number of patients in their charge |  |  |  |  |
| 0–4 | 0 |  |  |  |
| 5–9 | 0.77 | 0.29 | 1.26 | 0.001 |
| 10–14 | 0.71 | −0.06 | 1.50 | 0.072 |
| >15 | 1.08 | −0.27 | 2.45 | 0.117 |
| Unknown | −1.32 | −2.65 | −0.0006 | 0.049 |
| General medicine department rotation |  |  |  |  |
| Yes | 0 |  |  |  |
| No | −0.03 | −0.47 | 0.41 | 0.883 |
| Self-study time |  |  |  |  |
| None | 0 |  |  |  |
| 0–30 min per day | −0.22 | −1.32 | 0.87 | 0.683 |
| 31–60 min per day | 0.20 | −0.89 | 1.29 | 0.719 |
| 61 to 90 min per day | 0.76 | −0.37 | 1.91 | 0.188 |
| >91 min per day | 0.99 | −0.38 | 2.36 | 0.158 |
| Weekly duty hours |  |  |  |  |
| 0–59 h per week | 0 |  |  |  |
| 60–79 h per week | 0.73 | 0.30 | 1.15 | <0.001 |
| >80 h per week | −0.02 | −0.56 | 0.50 | 0.913 |
| Note: The four domains are: medical interview/professionalism, symptomatology/clinical reasoning, clinical procedure, and disease knowledge. | | | | |
